# PD54 Managed Entry Agreements: A Fad Or Potential Policy Option For Latin America? State Of The Art, Opportunities, And Challenges

**DOI:** 10.1017/S0266462325101864

**Published:** 2025-12-29

**Authors:** Hector Castro, Silvana Lay, Rabia Sucu

## Abstract

**Introduction:**

Countries face the challenge of advancing access to innovative health technologies. There is growing interest in managed entry agreements (MEAs) in Latin America, but very few publications. A recent cross-country comparison of Argentina, Brazil, Chile, Colombia, Costa Rica, Ecuador, Mexico, and Peru analyzed state-of-the-art MEAs in these settings. After collecting information from 69 relevant stakeholders through focus groups, interviews, and surveys, a series of recommendations for MEA scalability were provided.

**Methods:**

Qualitative methods were used. A desk review of recent regulations and publications of successful MEAs in the eight countries of interest was performed, followed by eight focus groups with 63 key informants. Also, three key informants from local authorities and three representatives from the local pharmaceutical federations of Argentina, Brazil, and Chile were invited to participate in a survey. The qualitative methods used served to provide relevant information to inform a set of recommendations.

**Results:**

In total, 69 key informants participated in the study. The level of knowledge and understanding of the use and limitations of MEAs was moderate to high in all countries. There seemed to be positive or moderate political will and policy commitment to implementing MEAs in the region.

**Conclusions:**

The most common barriers to implementing MEAs were a lack of clear rules, roles, and responsibilities; the lack of enabling legal and policy frameworks; and the scarcity of available information on successful implementation of MEAs in neighboring countries.

